# Surgical Treatment of Endometrial Cancer and Atypical Hyperplasia: A Trend Shift from Laparotomy to Laparoscopy

**DOI:** 10.1155/2011/829425

**Published:** 2011-07-13

**Authors:** Erik Qvigstad, Marit Lieng

**Affiliations:** Department of Gynecology, Oslo University Hospital Ullevål and University of Oslo, 0407 Oslo, Norway

## Abstract

*Background*. Laparoscopic hysterectomy has proved to be a safe alternative to open surgery in women with benign indications. Few studies compare laparotomy and laparoscopy in gynecologic oncology, and the objective of this study was to analyze the feasibility and development of laparoscopic surgery in endometrial cancer patients. *Material and Methods*. Records from all women having a hysterectomy due to premalignant or malignant endometrial changes during the years 2002–2009 were examined retrospectively. *Results*. A total of 521 hysterectomies were performed during the study period. Laparoscopy was performed in about 20% of the cases in the first two years, increasing to 83% in the last year of the period. Moreover, the laparoscopic technique was increasingly applied in older women, more obese women and in women with high-risk preoperative diagnosis, without increasing the complication rate. *Conclusions*. As for benign indications, laparoscopic hysterectomy in endometrial cancer patients should be preferred whenever possible.

## 1. Introduction

Endometrial cancer is the most common gynecological malignancy [1, 2]. The main treatment of early-stage endometrial cancer is surgery, including total hysterectomy, bilateral salping oophorectomy, and pelvic and aortic lymph-node dissection and removal if indicated [3]. Although total abdominal hysterectomy with vertical midline incision is still the standard treatment for early-stage endometrial cancer, laparoscopic approach has been in increasing use since Reich et al. published the first laparoscopic assisted vaginal hysterectomy in 1989 [4]. For benign indications, like fibroids and bleeding disorders, several prospective controlled studies have shown total laparoscopic hysterectomy as a safe alternative to open surgery [5, 6]. Endometrial cancer patients are, however, usually older, quite often obese, and a substantial number of them present with comorbidity at the time of surgery. The laparoscopy-related benefits observed in women with benign indications could, therefore, be reduced or different in these patients. A few randomized studies evaluating different aspects of laparoscopy versus laparotomy in patients with endometrial cancer have been published [7–13]. In a recent review by Hauspy et al., comparing laparoscopic approach with open surgery in endometrial cancer patients, the same benefits of laparoscopy were observed as for women with benign indication, and, based on currently available data, they recommend that women with endometrial cancer should be offered minimally invasive surgery as part of their treatment whenever possible [14].

We have previously reported on changing hysterectomy technique, mainly for benign indications [15]. We strongly believe that minimally invasive surgery is beneficial also for endometrial cancer patients, as was briefly mentioned in our previous report. Based on this philosophy, we have observed a marked trend shift from laparotomy to laparoscopy in our department, a university teaching hospital. Moreover, older and obese women are increasingly having the benefits of laparoscopy instead of open surgery.

## 2. Material and Methods

Following approval by the Regional Committee for Medical Research Ethics in Southern and Eastern Norway and Oslo University Hospital's Advisory Committee on the Protection of Patient Records, the medical records of all women who had had hysterectomy due to premalignant or malignant endometrial changes during the years 2002–2009 were examined retrospectively. For each year in the study period, the number of hysterectomies was recorded as well as women's age and body mass index (BMI), preoperative histological diagnosis, surgical technique (open abdominal or laparoscopic), duration of surgery, complications during the hospital stay, and postoperative histological diagnosis. During the study period of eight years, the surgeon decided the surgical approach based on present knowledge and experience, without any external pressure related to surgical approach. There was no randomization of the patients having surgery. The preoperative diagnosis was based on endometrial sampling by suction curette (Pipelle; Prodimed, Neuilly-en-Thelle, France) or D&C. In addition, all patients had an MRI examination of the pelvis preoperatively. In the laparotomy group, a vertical midline incision was used, followed by peritoneal washing and total hysterectomy. In the laparoscopy group, the procedure was also straightforward with four entrance ports, peritoneal washing, hysterectomy with vaginal delivery of the uterus, and laparoscopic closure of the vaginal cuff. Independent of the surgical technique, bilateral salpingoophorectomy and lymph node dissection were performed whenever indicated. The operative procedures were essentially similar, independent of surgeon and patient, even though the position of the surgeon and camera quite often was changed during laparoscopic paraaortal node dissection. Complications related to the surgical procedure were registered.

**Statistics**

Data were analyzed using SPSS for Windows (SPSS 17.0; SPSS, Inc. Chicago, Ill, USA). All statistical tests were performed two-sided, with a 5% significance level. Normally distributed continuous data from two groups were analyzed using a two-sided independent samples Student's *t*-test and when paired, the paired samples *t*-test. Categorical data were analyzed using Pearson's chi-square test.

Procedures commenced by laparoscopy but converted to laparotomy (*n* = 10) were included in the laparotomy group during the statistical analyses.

## 3. Results

A total of 521 hysterectomies were performed due to atypical endometrial hyperplasia and endometrial cancer during the study period from 2002 through 2009, varying between 51 and 86 procedures yearly. The mean age of the included women at the time of hysterectomy was 63.7 years (SD 11.8), with a mean BMI of 28.2 (SD 6.5). Preoperative endometrial biopsies were obtained from all the included women. As shown in Table 1, the preoperative histological diagnosis was endometrioid adenocarcinoma in 386 women (74.1%), atypical endometrial hyperplasia in 90 women (17.3%) and serous papillary and clear cell carcinoma in 23 (4.4%) and 22 women (4.2%), respectively. The preoperative degree of tumour differentiation in women with endometrioid adenocarcinoma was high tumour differentiation in 153 women, medium differentiation in 111 women, and low tumour differentiation in 42 women. The degree of tumour differentiation was unknown preoperatively in 80 of the women. 

**Figure 1:**
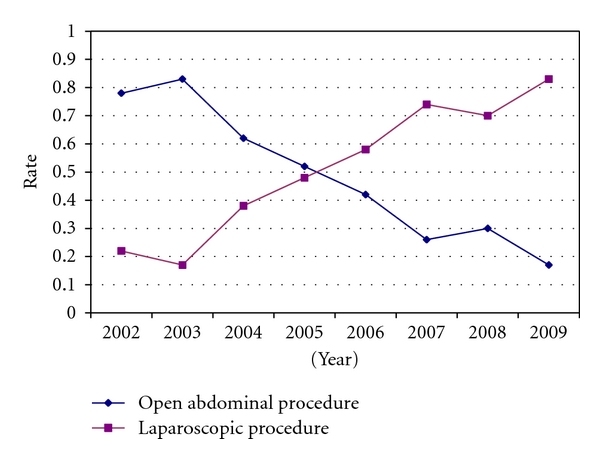
Rate of open abdominal and laparoscopic hysterectomy in women with endometrial cancer, 2002–2009.

During the study period, the hysterectomy was performed by an open abdominal approach in 230 women (44.1%) and by laparoscopy in 281 women (53.9%). In 10 women (1.9%), the procedure was commenced by laparoscopy but converted to laparotomy during the procedure. Open surgery dominated during the first years of the study period. Hence, laparoscopy was performed in 22% of the cases in 2002 and in 17% in 2003. From 2006, laparoscopy has been the most practised surgical procedure, and minimally invasive surgery has replaced open surgery, the last few years, with laparoscopic approach in 70% and 83% of the patients in 2008 and 2009, respectively. The considerable trend shift of surgical approach during the study period is illustrated in Figure 1.

**Figure 2:**
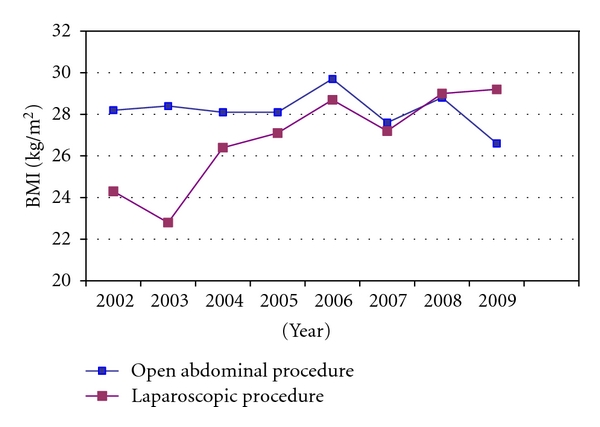
Mean body mass index (BMI) in women having open abdominal and laparoscopic procedures in 2002–2009.

**Figure 3:**
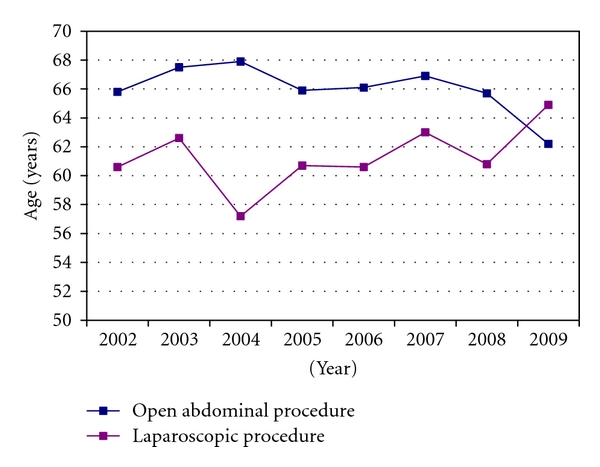
Mean age of women having laparoscopic and open abdominal procedures in 2002–2009.

In the early study period, women who were treated by laparoscopy had a lower BMI compared to women who underwent laparotomy. As illustrated in Figure 2, this has changed through the eight-year period, and women who had laparoscopic procedure in the last few years had higher BMI compared to the laparotomy group. Previously, advanced laparoscopic procedures were more unusual in older patients, and the average age of the included patients were almost 64 years. As observed for obesity, there has been a tendency with more laparoscopic procedures in older patients through the eight-year study period (Figure 3). A similar tendency was seen when preoperative histological diagnosis was examined in relation to the surgical approach. In 2002, all women with a high-risk preoperative histology (endometrioid adenocarcinoma with low tumour differentiation, clear cell, and serous papillary carcinoma) were treated by an open abdominal approach. Even though the laparoscopy procedures have varied among these patients through the years, a reasonable number of patients have experienced the benefits of minimally invasive surgery, and, in the last registered year (2009), two thirds of these patients had laparoscopic surgery. 

The operative time was significantly higher in laparoscopic compared with open procedures, 107 minutes (SD 29) and 87 minutes (SD 27), respectively (mean difference 20, 95% CI: 15.1, 24.8, *P* < 0.001). Mean hospital stay was 3.1 days (SD 1.9) for laparoscopic procedures and 8.4 days (SD 7.8) for open abdominal procedures (mean difference 5.3, 95% CI: 4.4, 6.3, *P* < 0.001). On average, women went back to normal activity 2-3 weeks after laparoscopy and 5-6 weeks after laparotomy. 

Complications related to the surgery are presented in Table 2. The incidence of injury of the bowels was significant higher in women treated by laparotomy compared to laparoscopy, 2.5% versus 0.4% (*P* = 0.033). Other major complications such as injury of the bladder were comparable in the two surgical approaches. There was no difference in complication rates from the first to the last years of the study period. The occurrence of postoperative wound infection was significantly higher in women treated by open abdominal approach compared to laparoscopic procedures, 10.4% and 0.4%, respectively (*P* < 0.001). Women who suffered from postoperative wound infections had significant higher BMI (mean BMI 31.0, SD 7.2) compared to women who did not have wound infections (mean BMI 27.9, SD 6.0) (mean difference 3.0, 95% CI 0.3, 5.8, *P* = 0.032). In addition, three women developed pulmonary embolus after the open abdominal procedure, a complication not observed in women who underwent laparoscopy.

## 4. Discussion

This retrospective study in a university hospital in Norway shows a remarkable trend shift from open surgery to laparoscopic hysterectomy in endometrial cancer patients. In the eight-year period, there were essentially the same senior consultants and consultants, without any external pressure, who changed from about 20% to over 80% laparoscopic procedures in this patient group.

Several studies, including the Gynecologic Oncology Group LAP2 randomized controlled trial, support laparoscopic surgery in these patients with less postoperative complications [16]. In recent years, many hospitals have invested in robotic units, especially in gynecologic oncology surgery, because of benefits including enhanced ergonomics, better surgeon comfort, and more easy performance of complicated surgery. In a cost comparison of surgical treatment of endometrial cancer, laparoscopy was the least costly approach, and in many aspects even robotic surgery, was less costly than hysterectomy by open surgery [17]. In our department, all laparoscopic procedures were performed without robotic units. Being a university teaching hospital, we teach and train younger doctors, and consequently we have laparoscopic surgeons on different experience levels, and the most experienced are involved in oncologic procedures. In our setting, we believe that robotic surgery has few advantages. 

During the study period of eight years, we have not only increased laparoscopic surgery, but we have included older and more obese women as well as all histopathologic grade I endometrial cancers. Although some have regarded obesity as a relative contraindication to operative laparoscopy, laparoscopic techniques have proved to be particularly well suited to the treatment of obese patients because of less postoperative complications [18]. The laparoscopic approach results in fewer operative complications, faster recovery, shorter hospital stay, and less pain compared with open procedures. Extensive laparotomy procedures increase the risk of pneumonia, deep venous thrombosis, ileus, and wound infections, leading to increased postoperative morbidity and possibly mortality and longer hospital stays [19–22].

In the first years with laparoscopic approach to endometrial cancer patients, we preferably performed the operative procedure in patients with histological proven stage I endometrial adenocarcinoma or complex atypical hyperplasia. With increasing experience, we now perform laparoscopy also in nonendometrial carcinoma types where pelvic and aorta lymph node dissection is indicated. Especially in obese patients and when lymph node dissection is part of the surgical procedure, many surgeons prefer robotic surgery. Robotic surgery will probably grow in popularity and use in the treatment of endometrial cancer, but the costs associated with the robotic surgery are the limitations in many hospitals and countries [17]. 

Minimal invasive surgery has many advantages for the patient, the hospital and the society. As for benign indications, laparoscopic hysterectomy in endometrial cancer patients should be preferred whenever possible [14]. In our department, we have on the basis of knowledge and experience observed a marked trend shift from open surgery to laparoscopy in the last decade. This has been obtained within the same group of gynecologic surgeons, without any external pressure. According to the surgeons in our department, the difference in recovery after surgery was essential for the observed trend shift in surgical technique throughout the time period. We have increased the laparoscopic approach from about 20% to over 80% in just a few years. Although long-term outcomes were not evaluated in this study, the enhanced laparoscopic activity is very promising for women suffering from endometrial cancer as well as for minimal invasive surgery around the world.

## 5. Conclusions

As for benign indications, total laparoscopic hysterectomy is a safe alternative to open surgery in endometrial cancer patients, and the laparoscopic approach results in fewer operative complications, faster recovery, shorter hospital stay, and less pain. During an eight-year period from 2002 to 2009, we have had a complete trend shift from about 20% laparoscopic procedures in this patient group to about 80% being treated with minimal invasive technique. Moreover, laparoscopy was increasingly applied in older and more obese patients, and we conclude that laparoscopic hysterectomy should be preferred whenever possible in endometrial cancer patients.

## Figures and Tables

**Table 1:** Preoperative histological diagnosis.

Preoperative histological diagnosis	Open abdominal procedure *n* (%)	Laparoscopic procedure *n* (%)	Total *n* (%)
Endometrioid adenocarcinoma	176 (73.6)	210 (74.5)	386 (74.1)
Atypical endometrial hyperplasia	25 (10.5)	65 (23.0)	90 (17.3)
Serous papillar carcinoma	17 (7.1)	6 (2.1)	23 (4.4)
Clear cell carcinoma	21 (8.8)	1 (0.4)	22 (4.2)
Total	240	281	521 (100)

**Table 2:** Complications in women treated by open abdominal and laparoscopic procedures.

	Open abdominal procedure *n* (%)	Laparoscopic procedure *n* (%)
Injury to the bowels	6 (2.5)	1 (0.4)
Injury to the bladder	1 (0.4)	2 (0.7)
Postoperative infection	25 (10.4)	1 (0.4)
Pulmonary embolus	3 (1.3)	0
Other	38 (15.8)	24 (8.5)
